# Unusual hemiacetal structure derived from Salvinorin A

**DOI:** 10.1107/S160053680800144X

**Published:** 2008-06-28

**Authors:** Paulo Carvalho, Ruslan Bikbulatov, Jordan K. Zjawiony, Mitchell A. Avery

**Affiliations:** a Department of Medicinal Chemistry, University of Mississippi, 417 Faser Hall, University, MS 38677, USA; b Department of Pharmacognosy, University of Mississippi, PO Box 1848, 443 Faser Hall, University, MS, 38677-1848, USA; c National Center for Natural Products Research, Research Institute of Pharmaceutical Sciences, School of Pharmacy, University of Mississippi, University, MS 38677, USA; d Department of Chemistry and Biochemistry, University of Mississippi, University, MS 38677, USA

## Abstract

The salvinorin A analog dimethyl (2*R*,3a*R*,4*R*,6a*R*,7*R*,9*S*,9a*S*,9b*S*)-2-(3-fur­yl)-9,9a-dihydr­oxy-3a,6a-dimethyl­dodeca­hydro­benzo[*de*]chromene-4,7-dicarboxyl­ate, C_22_H_30_O_8_, has a relatively simple spatial arrangement in which mol­ecules are linked into layers by two pairs of O—H⋯O hydrogen bonds. Each mol­ecule has as the central feature a dodeca­hydro-1*H*-phenalene ring system. Its three six-membered rings are in the chair conformation, with two axial methyl groups, one axial OH, and one equatorial OH, these OH groups being directly responsible for linking of the mol­ecules in the crystal structure.

## Related literature

For the synthesis of analogs of salvinorin A, see: Bikbulatov *et al.* (2007 ▶); Lee *et al.* (2006 ▶); Beguin *et al.* (2006 ▶); Stewart *et al.* (2006 ▶) and references cited therein. For modifications of salvinorin A with changed pharmacological profile, see: Rothman *et al.* (2007 ▶); Groer *et al.* (2007 ▶); Tidgewell *et al.* (2006 ▶); Harding *et al.* (2005 ▶, 2006 ▶).
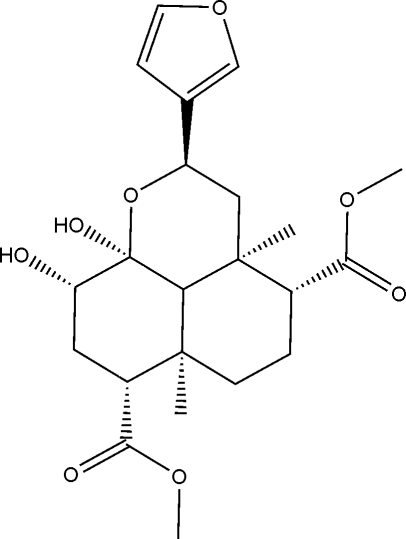


## Experimental

### 

#### Crystal data

C_22_H_30_O_8_
*M* *_r_* = 422.46
Monoclinic, 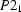

*a* = 11.6801 (5) Å
*b* = 6.0522 (3) Å
*c* = 15.3739 (6) Å
β = 107.678 (2)°
*V* = 1035.47 (8) Å^3^
*Z* = 2
Cu *K*α radiation
μ = 0.86 mm^−1^
*T* = 296 (2) K
0.32 × 0.15 × 0.13 mm

#### Data collection

Bruker SMART CCD area-detector diffractometer
Absorption correction: none
18724 measured reflections
3726 independent reflections
3615 reflections with *I* > 2σ(*I*)
*R*_int_ = 0.037

#### Refinement

*R*[*F* ^2^ > 2σ(*F* ^2^)] = 0.037
*wR*(*F* ^2^) = 0.096
*S* = 1.05
3726 reflections
277 parameters
1 restraint
H-atom parameters constrained
Δρ_max_ = 0.41 e Å^−3^
Δρ_min_ = −0.21 e Å^−3^
Absolute structure: Flack (1983 ▶), 1587 Friedel pairs
Flack parameter: 0.14 (18)

### 

Data collection: *SMART* (Bruker, 2003 ▶); cell refinement: *SAINT* (Bruker, 2003 ▶); data reduction: *SAINT*; program(s) used to solve structure: *SHELXS97* (Sheldrick, 2008 ▶); program(s) used to refine structure: *SHELXL97* (Sheldrick, 2008 ▶); molecular graphics: *SHELXTL* (Sheldrick, 2008 ▶); software used to prepare material for publication: *SHELXTL*.

## Supplementary Material

Crystal structure: contains datablocks I, global. DOI: 10.1107/S160053680800144X/gw2037sup1.cif

Structure factors: contains datablocks I. DOI: 10.1107/S160053680800144X/gw2037Isup2.hkl

Additional supplementary materials:  crystallographic information; 3D view; checkCIF report

## Figures and Tables

**Table 1:** Hydrogen-bond geometry (Å, °)

*D*—H⋯*A*	*D*—H	H⋯*A*	*D*⋯*A*	*D*—H⋯*A*
O3—H3⋯O2^i^	0.82	1.99	2.757 (2)	155
O2—H2*A*⋯O1^i^	0.82	2.07	2.787 (2)	146

Symmetry code: (i) 
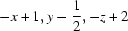
.

**Fig. 1.**
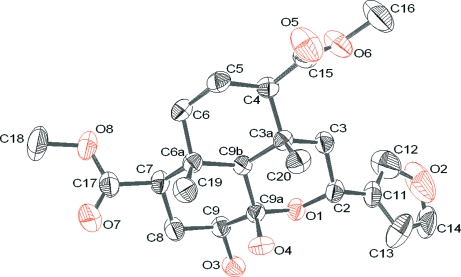
Molecular projection, showing the atom-labeling scheme. H atoms were ommited to allow better visualization.

**Fig. 2.**
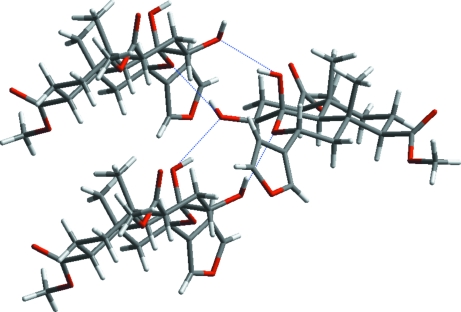
The packing in the crystal structure, showing the O—H···O hydrogen bonds as dashed blue lines.

**Fig. 3.**
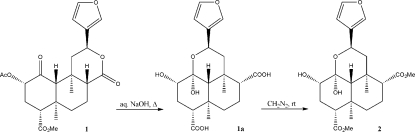
Synthesis of the title compound

## supplementary crystallographic information

### Comment

.

The structure was solved using Direct methods and difference Fourier techniques *SHELXTL*, V6.12 (Sheldrick, 2008). Hydrogen atoms were placed in their expected chemical positions using the HFIX command and were included in the final cycles of least squares with isotropic *U^ij^* related to the atoms ridden upon. All non-hydrogen atoms were refined anisotropically.Structure solution, refinement, graphics and generation of publication materials were performed by using *SHELXTL*, V6.12 software. Additional details of data collection and structure refinement are given in Table 1.

### Experimental

Synthesis of hemiacetal (2): Salvinorin A (10 mg, 23 mmol) was placed in aqueous 5% KOH (5 ml) and refluxed for two hours producing a yellow solution. Upon reaching room temperature, the solution was cooled in an ice bath and neutralized with cold aqueous 0.5*M* HCl. The resulting precipitate was collected by vacuum filtration. The product was purified by passing through a short silica column eluting with ethyl acetate to yield 6.2 mg of 1a (69%).

1 ml of TMSCHN_2_ (0.13 mmol) in benzene was added at room temperature to a solution of 1a (20 mg, 0.05 mmol) in methanol (5 ml). The mixture was stirred at room temperature for 30 min and concentrated to give the corresponding dimethyl ester 2. The product was purified by column chromatography using hexanes: ethyl acetate (2:1) for elution. Yield 18.3 mg (87%).

Crystals of dimethyl (2*R*,3a*R*,4*R*,6a*R*,7*R*,9*S*,9a*S*,9 b*S*)-2-(3-furyl)-9,9a-dihydroxy-3a,6a-dimethyldodecahydrobenzo[*de*]chromene-4,7-dicarboxylate (2) were obtained from slow evaporation of a solution in ethyl acetate/hexanes 1:9. A suitable crystal was coated with Paratone N oil, suspended in a CryoLoop (Hampton Research) and placed in a cooled nitrogen gas stream at 100 K on a Bruker D8 *APEX* II CCD sealed tube diffractometer with graphite monochromated Cu *K*_a _(1.54178 Å) radiation. Data were measured using a series of combinations of phi and omega scans with 10 s frame exposures and 0.5^o ^frame widths. Data collection, indexing and initial cell refinements were all carried out using *APEX* II software (Bruker, 2003).Frame integration and final cell refinements were done using *SAINT *(Bruker, 2003) software. The final cell parameters were determined from least-squares refinement on 3446 reflections

Considering that the Flack parameter (Flack, 1983) does not confirm unambiguously the absolute configuration of the molecule, The chiral centers were assigned based in the original known configuration of the starting material, Salvinorin A.

### Refinement

All H atoms were located in difference maps and treated as riding atoms, with the following distance restraints: C—H = 0.93 Å, *U*_iso_=1.2Ueq (C) for Csp2, C—H = 0.98 Å, *U*_iso_ = 1.2Ueq (C) for CH, C—H = 0.97 Å, *U*_iso_ = 1.2Ueq (C) for CH2, C—H = 0.96 Å, *U*_iso_ = 1.5Ueq (C) for CH3, O—H = 0.82 Å, *U*_iso_ = 1.5Ueq (O) for OH.

### Crystal data

**Table:**

C_22_H_30_O_8_	*F*_000_ = 452
*M**_r_* = 422.46	*D*_x_ = 1.355 Mg m^−^^3^
Monoclinic, *P*2_1_	Cu *K*α radiation λ = 1.54178 Å
Hall symbol: P 2yb	Cell parameters from 8551 reflections
*a* = 11.6801 (5) Å	θ = 3.0–69.4º
*b* = 6.0522 (3) Å	µ = 0.86 mm^−^^1^
*c* = 15.3739 (6) Å	*T* = 296 (2) K
β = 107.678 (2)º	Blocks, colourless
*V* = 1035.47 (8) Å^3^	0.32 × 0.15 × 0.13 mm
*Z* = 2	

### Data collection

**Table:**

Bruker SMART CCD area-detector diffractometer	3615 reflections with *I* > 2σ(*I*)
Monochromator: graphite	*R*_int_ = 0.037
*T* = 100 K	θ_max_ = 69.4º
phi and ω scans	θ_min_ = 3.0º
Absorption correction: none	*h* = −13→13
18724 measured reflections	*k* = −7→7
3726 independent reflections	*l* = −18→18

### Refinement

**Table:**

Refinement on *F*^2^	Hydrogen site location: inferred from neighbouring sites
Least-squares matrix: full	H-atom parameters constrained
*R*[*F*^2^ > 2σ(*F*^2^)] = 0.037	*w* = 1/[σ^2^(*F*_o_^2^) + (0.0458*P*)^2^ + 0.4492*P*] where *P* = (*F*_o_^2^ + 2*F*_c_^2^)/3
*wR*(*F*^2^) = 0.096	(Δ/σ)_max_ < 0.001
*S* = 1.05	Δρ_max_ = 0.42 e Å^−^^3^
3726 reflections	Δρ_min_ = −0.21 e Å^−^^3^
277 parameters	Extinction correction: none
1 restraint	Absolute structure: Flack (1983), 1587 Friedel pairs
Primary atom site location: structure-invariant direct methods	Flack parameter: 0.14 (18)
Secondary atom site location: difference Fourier map	

### Special details

**Table:**

Experimental. The structure was solved using Direct methods and difference Fourier techniques *SHELXTL*, V6.12 (Bruker, 2003). Hydrogen atoms were placed in their expected chemical positions using the HFIX command and were included in the final cycles of least squares with isotropic *U^ij^* related to the atoms ridden upon. All non-hydrogen atoms were refined anisotropically.Structure solution, refinement, graphics and generation of publication materials were performed by using *SHELXTL*, V6.12 software. Additional details of data collection and structure refinement are given in Table 1.
Geometry. All e.s.d.'s (except the e.s.d. in the dihedral angle between two l.s. planes)are estimated using the full covariance matrix. The cell e.s.d.'s are takeninto account individually in the estimation of e.s.d.'s in distances, anglesand torsion angles; correlations between e.s.d.'s in cell parameters are onlyused when they are defined by crystal symmetry. An approximate (isotropic)treatment of cell e.s.d.'s is used for estimating e.s.d.'s involving l.s. planes.
Refinement. Refinement of *F*^2^ against ALL reflections. The weighted *R*-factor *wR* andgoodness of fit *S* are based on *F*^2^, conventional *R*-factors *R* are basedon *F*, with *F* set to zero for negative *F*^2^. The threshold expression of*F*^2^ > σ(*F*^2^) is used only for calculating *R*-factors(gt) *etc*. and isnot relevant to the choice of reflections for refinement. *R*-factors basedon *F*^2^ are statistically about twice as large as those based on *F*, and *R*-factors based on ALL data will be even larger.

### Fractional atomic coordinates and isotropic or equivalent isotropic displacement parameters (Å^2^)

**Table:**

	*x*	*y*	*z*	*U*_iso_*/*U*_eq_	
C2	0.18522 (17)	0.9011 (4)	0.91971 (13)	0.0308 (5)	
H2	0.1571	0.7482	0.9184	0.037*	
C5	0.15034 (17)	0.9159 (3)	0.58226 (12)	0.0226 (4)	
H5A	0.1171	0.7690	0.5668	0.027*	
H5B	0.1226	1.0076	0.5282	0.027*	
C3	0.12566 (17)	1.0070 (4)	0.82782 (12)	0.0261 (4)	
H3A	0.1552	1.1568	0.8281	0.031*	
H3B	0.0396	1.0144	0.8176	0.031*	
C6	0.28737 (16)	0.9033 (3)	0.61198 (12)	0.0217 (4)	
H6A	0.3195	1.0521	0.6224	0.026*	
H6B	0.3122	0.8401	0.5626	0.026*	
C4	0.10549 (16)	1.0123 (3)	0.65832 (12)	0.0209 (4)	
H4	0.1380	1.1622	0.6711	0.025*	
C8	0.54094 (17)	0.7152 (4)	0.82950 (13)	0.0283 (4)	
H8A	0.5286	0.5568	0.8302	0.034*	
H8B	0.6268	0.7428	0.8472	0.034*	
C9	0.48763 (17)	0.8258 (3)	0.89657 (12)	0.0256 (4)	
H9	0.4971	0.9857	0.8914	0.031*	
C7	0.48158 (17)	0.8048 (3)	0.73302 (12)	0.0230 (4)	
H7	0.4950	0.9648	0.7349	0.028*	
C3A	0.15070 (16)	0.8761 (3)	0.74901 (11)	0.0202 (4)	
C6A	0.34211 (16)	0.7655 (3)	0.69886 (12)	0.0197 (4)	
C9A	0.35251 (18)	0.7770 (3)	0.87112 (13)	0.0239 (4)	
C9B	0.29098 (16)	0.8644 (3)	0.77341 (11)	0.0191 (4)	
H9B	0.3154	1.0198	0.7767	0.023*	
C10	0.16237 (19)	1.0186 (5)	0.99865 (14)	0.0448 (7)	
C12	0.1441 (3)	1.0759 (8)	1.13703 (17)	0.0705 (12)	
H12	0.1433	1.0539	1.1967	0.085*	
C11	0.1675 (3)	0.9226 (8)	1.0830 (2)	0.0778 (12)	
H11	0.1846	0.7751	1.0985	0.093*	
C13	0.1359 (3)	1.2334 (7)	1.00599 (18)	0.0751 (12)	
H13	0.1281	1.3414	0.9615	0.090*	
C16	−0.03024 (17)	1.0299 (3)	0.62641 (12)	0.0252 (4)	
C18	0.54524 (17)	0.7069 (4)	0.66942 (13)	0.0261 (4)	
C14	0.08432 (17)	0.6535 (3)	0.73785 (13)	0.0257 (4)	
H14A	0.1270	0.5548	0.7856	0.039*	
H14B	0.0801	0.5906	0.6796	0.039*	
H14C	0.0045	0.6763	0.7414	0.039*	
C15	0.31545 (17)	0.5190 (3)	0.67784 (13)	0.0259 (4)	
H15A	0.2316	0.5000	0.6460	0.039*	
H15B	0.3359	0.4370	0.7339	0.039*	
H15C	0.3623	0.4661	0.6405	0.039*	
C19	0.6056 (3)	0.7648 (5)	0.53744 (18)	0.0490 (7)	
H19A	0.6898	0.7427	0.5672	0.073*	
H19B	0.5951	0.8701	0.4890	0.073*	
H19C	0.5692	0.6270	0.5128	0.073*	
C17	−0.1957 (2)	1.2172 (6)	0.65095 (18)	0.0505 (7)	
H17A	−0.2322	1.2419	0.5868	0.076*	
H17B	−0.2122	1.3404	0.6846	0.076*	
H17C	−0.2279	1.0848	0.6688	0.076*	
O1	0.31411 (11)	0.9003 (3)	0.93685 (8)	0.0273 (3)	
O3	0.32773 (13)	0.5526 (2)	0.87711 (9)	0.0286 (3)	
H3	0.3709	0.5030	0.9256	0.043*	
O5	−0.09773 (12)	0.9134 (3)	0.57030 (10)	0.0352 (4)	
O6	−0.06776 (13)	1.1939 (3)	0.66985 (10)	0.0349 (4)	
O2	0.54549 (13)	0.7663 (3)	0.98856 (9)	0.0311 (3)	
H2A	0.5935	0.6663	0.9902	0.047*	
O7	0.58818 (15)	0.5249 (3)	0.67619 (11)	0.0429 (4)	
O8	0.54884 (15)	0.8474 (3)	0.60324 (11)	0.0383 (4)	
O4	0.1223 (2)	1.2643 (7)	1.09199 (17)	0.1192 (16)	

### Atomic displacement parameters (Å^2^)

**Table:**

	*U*^11^	*U*^22^	*U*^33^	*U*^12^	*U*^13^	*U*^23^
C2	0.0193 (9)	0.0538 (13)	0.0210 (9)	−0.0075 (10)	0.0087 (7)	−0.0039 (9)
C5	0.0235 (9)	0.0264 (9)	0.0172 (8)	−0.0001 (8)	0.0053 (7)	0.0016 (7)
C3	0.0159 (9)	0.0401 (11)	0.0237 (9)	−0.0026 (8)	0.0078 (7)	−0.0048 (9)
C6	0.0233 (9)	0.0242 (9)	0.0195 (8)	−0.0030 (8)	0.0093 (7)	−0.0008 (7)
C4	0.0202 (9)	0.0206 (9)	0.0226 (9)	−0.0014 (7)	0.0074 (7)	−0.0020 (7)
C8	0.0190 (9)	0.0402 (12)	0.0239 (9)	0.0031 (8)	0.0037 (8)	−0.0023 (8)
C9	0.0222 (10)	0.0362 (11)	0.0178 (8)	−0.0017 (8)	0.0051 (7)	−0.0020 (8)
C7	0.0198 (9)	0.0281 (10)	0.0215 (9)	−0.0020 (8)	0.0067 (7)	−0.0046 (7)
C3A	0.0161 (9)	0.0257 (9)	0.0185 (8)	−0.0028 (7)	0.0050 (7)	−0.0016 (7)
C6A	0.0174 (9)	0.0236 (9)	0.0180 (8)	−0.0013 (7)	0.0054 (7)	−0.0011 (7)
C9A	0.0222 (10)	0.0311 (10)	0.0199 (8)	−0.0026 (8)	0.0084 (7)	0.0001 (8)
C9B	0.0174 (9)	0.0207 (9)	0.0190 (8)	−0.0034 (7)	0.0053 (7)	−0.0003 (7)
C10	0.0168 (10)	0.098 (2)	0.0218 (10)	−0.0058 (12)	0.0089 (8)	−0.0109 (12)
C12	0.0373 (15)	0.160 (4)	0.0190 (11)	0.0052 (19)	0.0150 (10)	−0.0089 (18)
C11	0.082 (2)	0.126 (3)	0.0380 (15)	−0.036 (2)	0.0370 (15)	−0.0145 (18)
C13	0.072 (2)	0.120 (3)	0.0285 (13)	0.050 (2)	0.0076 (13)	−0.0206 (16)
C16	0.0219 (9)	0.0290 (10)	0.0249 (9)	0.0025 (8)	0.0075 (8)	0.0039 (8)
C18	0.0175 (9)	0.0341 (11)	0.0254 (9)	0.0010 (8)	0.0048 (7)	−0.0053 (8)
C14	0.0209 (9)	0.0306 (10)	0.0243 (9)	−0.0062 (8)	0.0048 (7)	0.0024 (8)
C15	0.0252 (10)	0.0257 (10)	0.0254 (9)	0.0003 (8)	0.0060 (8)	−0.0028 (8)
C19	0.0518 (16)	0.0625 (17)	0.0461 (13)	0.0102 (13)	0.0350 (12)	0.0024 (13)
C17	0.0283 (11)	0.0711 (18)	0.0494 (14)	0.0197 (12)	0.0077 (11)	−0.0069 (13)
O1	0.0180 (7)	0.0454 (8)	0.0191 (6)	−0.0045 (6)	0.0066 (5)	−0.0041 (6)
O3	0.0278 (7)	0.0308 (7)	0.0235 (6)	−0.0033 (6)	0.0022 (5)	0.0080 (6)
O5	0.0237 (7)	0.0461 (9)	0.0313 (7)	−0.0011 (7)	0.0015 (6)	−0.0102 (7)
O6	0.0259 (7)	0.0379 (8)	0.0393 (8)	0.0089 (7)	0.0074 (6)	−0.0058 (7)
O2	0.0292 (8)	0.0361 (8)	0.0244 (7)	0.0024 (6)	0.0027 (6)	−0.0009 (6)
O7	0.0443 (9)	0.0492 (10)	0.0389 (8)	0.0204 (8)	0.0181 (7)	0.0006 (8)
O8	0.0409 (9)	0.0458 (10)	0.0395 (8)	0.0042 (7)	0.0290 (7)	0.0025 (7)
O4	0.0566 (14)	0.244 (5)	0.0456 (13)	0.063 (2)	−0.0011 (11)	−0.063 (2)

### Geometric parameters (Å, °)

**Table:**

C2—O1	1.447 (2)	C9A—O1	1.433 (2)
C2—C10	1.499 (3)	C9A—C9B	1.548 (2)
C2—C3	1.515 (3)	C9B—H9B	0.9800
C2—H2	0.9800	C10—C13	1.349 (5)
C5—C6	1.527 (3)	C10—C11	1.405 (4)
C5—C4	1.535 (2)	C12—O4	1.318 (6)
C5—H5A	0.9700	C12—C11	1.328 (5)
C5—H5B	0.9700	C12—H12	0.9300
C3—C3A	1.548 (3)	C11—H11	0.9300
C3—H3A	0.9700	C13—O4	1.391 (3)
C3—H3B	0.9700	C13—H13	0.9300
C6—C6A	1.540 (2)	C16—O5	1.204 (2)
C6—H6A	0.9700	C16—O6	1.342 (3)
C6—H6B	0.9700	C18—O7	1.202 (3)
C4—C16	1.514 (2)	C18—O8	1.336 (3)
C4—C3A	1.567 (2)	C14—H14A	0.9600
C4—H4	0.9800	C14—H14B	0.9600
C8—C9	1.513 (3)	C14—H14C	0.9600
C8—C7	1.533 (3)	C15—H15A	0.9600
C8—H8A	0.9700	C15—H15B	0.9600
C8—H8B	0.9700	C15—H15C	0.9600
C9—O2	1.416 (2)	C19—O8	1.456 (3)
C9—C9A	1.535 (3)	C19—H19A	0.9600
C9—H9	0.9800	C19—H19B	0.9600
C7—C18	1.517 (3)	C19—H19C	0.9600
C7—C6A	1.571 (2)	C17—O6	1.440 (3)
C7—H7	0.9800	C17—H17A	0.9600
C3A—C14	1.538 (3)	C17—H17B	0.9600
C3A—C9B	1.568 (2)	C17—H17C	0.9600
C6A—C15	1.538 (3)	O3—H3	0.8200
C6A—C9B	1.563 (2)	O2—H2A	0.8200
C9A—O3	1.398 (2)		
			
O1—C2—C10	106.57 (15)	O3—C9A—O1	110.13 (15)
O1—C2—C3	109.20 (15)	O3—C9A—C9	112.80 (17)
C10—C2—C3	114.2 (2)	O1—C9A—C9	103.77 (15)
O1—C2—H2	108.9	O3—C9A—C9B	110.57 (15)
C10—C2—H2	108.9	O1—C9A—C9B	110.73 (15)
C3—C2—H2	108.9	C9—C9A—C9B	108.67 (15)
C6—C5—C4	111.12 (14)	C9A—C9B—C6A	114.36 (15)
C6—C5—H5A	109.4	C9A—C9B—C3A	113.02 (14)
C4—C5—H5A	109.4	C6A—C9B—C3A	116.49 (14)
C6—C5—H5B	109.4	C9A—C9B—H9B	103.6
C4—C5—H5B	109.4	C6A—C9B—H9B	103.6
H5A—C5—H5B	108.0	C3A—C9B—H9B	103.6
C2—C3—C3A	111.77 (17)	C13—C10—C11	105.5 (3)
C2—C3—H3A	109.3	C13—C10—C2	128.8 (3)
C3A—C3—H3A	109.3	C11—C10—C2	125.6 (3)
C2—C3—H3B	109.3	O4—C12—C11	108.6 (3)
C3A—C3—H3B	109.3	O4—C12—H12	125.7
H3A—C3—H3B	107.9	C11—C12—H12	125.7
C5—C6—C6A	114.07 (15)	C12—C11—C10	109.2 (4)
C5—C6—H6A	108.7	C12—C11—H11	125.4
C6A—C6—H6A	108.7	C10—C11—H11	125.4
C5—C6—H6B	108.7	C10—C13—O4	107.7 (3)
C6A—C6—H6B	108.7	C10—C13—H13	126.1
H6A—C6—H6B	107.6	O4—C13—H13	126.1
C16—C4—C5	110.25 (14)	O5—C16—O6	123.23 (18)
C16—C4—C3A	111.23 (15)	O5—C16—C4	125.74 (18)
C5—C4—C3A	112.15 (15)	O6—C16—C4	111.03 (16)
C16—C4—H4	107.7	O7—C18—O8	122.7 (2)
C5—C4—H4	107.7	O7—C18—C7	125.1 (2)
C3A—C4—H4	107.7	O8—C18—C7	112.15 (17)
C9—C8—C7	110.12 (16)	C3A—C14—H14A	109.5
C9—C8—H8A	109.6	C3A—C14—H14B	109.5
C7—C8—H8A	109.6	H14A—C14—H14B	109.5
C9—C8—H8B	109.6	C3A—C14—H14C	109.5
C7—C8—H8B	109.6	H14A—C14—H14C	109.5
H8A—C8—H8B	108.2	H14B—C14—H14C	109.5
O2—C9—C8	113.43 (16)	C6A—C15—H15A	109.5
O2—C9—C9A	110.34 (15)	C6A—C15—H15B	109.5
C8—C9—C9A	110.20 (15)	H15A—C15—H15B	109.5
O2—C9—H9	107.5	C6A—C15—H15C	109.5
C8—C9—H9	107.5	H15A—C15—H15C	109.5
C9A—C9—H9	107.5	H15B—C15—H15C	109.5
C18—C7—C8	108.61 (16)	O8—C19—H19A	109.5
C18—C7—C6A	112.77 (14)	O8—C19—H19B	109.5
C8—C7—C6A	112.85 (15)	H19A—C19—H19B	109.5
C18—C7—H7	107.4	O8—C19—H19C	109.5
C8—C7—H7	107.4	H19A—C19—H19C	109.5
C6A—C7—H7	107.4	H19B—C19—H19C	109.5
C14—C3A—C3	109.09 (16)	O6—C17—H17A	109.5
C14—C3A—C4	109.84 (14)	O6—C17—H17B	109.5
C3—C3A—C4	109.53 (15)	H17A—C17—H17B	109.5
C14—C3A—C9B	116.24 (16)	O6—C17—H17C	109.5
C3—C3A—C9B	105.55 (14)	H17A—C17—H17C	109.5
C4—C3A—C9B	106.38 (14)	H17B—C17—H17C	109.5
C15—C6A—C6	109.81 (15)	C9A—O1—C2	113.84 (14)
C15—C6A—C9B	115.31 (16)	C9A—O3—H3	109.5
C6—C6A—C9B	106.15 (14)	C16—O6—C17	116.61 (17)
C15—C6A—C7	109.91 (15)	C9—O2—H2A	109.5
C6—C6A—C7	108.86 (15)	C18—O8—C19	115.88 (19)
C9B—C6A—C7	106.57 (13)	C12—O4—C13	108.9 (3)

### Hydrogen-bond geometry (Å, °)

**Table:**

*D*—H···*A*	*D*—H	H···*A*	*D*···*A*	*D*—H···*A*
O3—H3···O2^i^	0.82	1.99	2.757 (2)	155
O2—H2A···O1^i^	0.82	2.07	2.787 (2)	146

Symmetry codes: (i) −*x*+1, *y*−1/2, −*z*+2.
